# Improved Cardiometabolic Health with Uterine-Preserving Fibroid Treatment Compared to Hysterectomy

**DOI:** 10.3390/jcm15051960

**Published:** 2026-03-04

**Authors:** Rachel Michel, Gregory W. Kirschen, Caitlin S. Stukel, Sydney L. Olson, Lisa Yanek, Katie Cameron, Wendy L. Bennett, Mostafa A. Borahay

**Affiliations:** 1 Department of Gynecology & Obstetrics, Johns Hopkins University, Baltimore, MD 21287, USA; rachel.michel.med@dartmouth.edu (R.M.); solson17@jh.edu (S.L.O.); katie.cameron@jhmi.edu (K.C.); 2 Geisel School of Medicine, Dartmouth College, Hanover, NH 03755, USA; caitlin.s.stukel.med@dartmouth.edu; 3 Department of Obstetrics & Gynecology, University of Pennsylvania, Philadelphia, PA 19104, USA; kirscheg@pennmedicine.upenn.edu; 4 Department of Internal Medicine, Johns Hopkins University, Baltimore, MD 21287, USA; lryanek@jhmi.edu (L.Y.); wendy.bennett@jhmi.edu (W.L.B.)

**Keywords:** cardiometabolic health, hysterectomy, leiomyoma, myomectomy, uterine fibroids

## Abstract

**Background/Objectives:** Over 600,000 hysterectomies are performed in the United States annually, with uterine fibroids being the most common indication. It remains unknown whether removal of the uterus is associated with poor cardiometabolic outcomes. This study aimed to determine whether hysterectomy is associated with adverse cardiometabolic outcomes among patients with uterine fibroids (myomectomy and no surgery as controls). **Methods:** Retrospective cohort study utilizing MarketScan^®^ Database of U.S. healthcare claims dataset, including patients aged 18–55 with uterine fibroid diagnosis in 2010 or 2011 and 10 years of continuous enrollment. Patients were stratified into three groups: hysterectomy, myomectomy, and no surgery. Patients were then followed for 10 years to determine incidence of cardiometabolic disorders. The main outcome measures included coronary artery disease, congestive heart failure, cardiac arrhythmia, stroke, hypertension, hyperlipidemia, type II diabetes, and peripheral artery disease, which were defined using the ICD-9, ICD-10, and CPT codes. **Results:** 34,722 participants with uterine fibroids were identified. Among these, 8,196 (23.61%) patients underwent hysterectomy, 1351 patients (3.89%) underwent myomectomy, and 25,175 (72.50%) patients did not undergo surgery. Hysterectomy was associated with higher age-adjusted odds of developing stroke (aOR = 1.11), hypertension (aOR = 1.14), hyperlipidemia (aOR = 1.14), and type II diabetes (aOR = 1.20) compared to no surgery. Hysterectomy was associated with higher age-adjusted odds of developing hyperlipidemia (aOR = 1.26) compared to myomectomy. Hysterectomy with removal of ovaries versus hysterectomy with retention of ovaries had similar cardiometabolic outcomes. **Conclusions:** Among patients with fibroids, hysterectomy was associated with higher odds of developing adverse cardiometabolic outcomes compared to myomectomy or no surgery. Retention of the ovaries was not protective among those undergoing hysterectomy.

## 1. Introduction

Hysterectomy is the second most common surgical procedure performed in women [1]. Of the 600,000 hysterectomies that occur in the US annually, more than half are performed for uterine fibroids, benign uterine tumors that cause symptoms such as heavy bleeding, pelvic pain, and infertility that affect up to 70% of the population [1,2,3]. The decision of whether to remove the ovaries at the time of a benign hysterectomy is guided by several factors, including patient age, concomitant pathology, and discussion with the patient regarding risk reduction for ovarian cancer. Removal of the ovaries prior to menopause is associated with worse long-term cardiovascular health and increased all-cause mortality; thus, the decision to perform bilateral oophorectomy at the time of benign hysterectomy is not taken lightly [4]. The Society for Academic Specialists in General Obstetrics and Gynecology advises against the routine removal of normal-appearing ovaries at the time of hysterectomy for patients up until age 65 [5].

The uterus has widely been considered inert regarding cardiometabolic health. However, accumulating evidence demonstrates that the uterus participates in vascular and metabolic signaling that may impact women’s long-term health beyond its reproductive function [6,7,8]. On the one hand, uterine fibroids are strongly associated with markers of poor cardiometabolic health, including obesity, hypertension, hyperlipidemia, and type 2 diabetes, while on the other hand, there is also evidence to suggest that in the absence of fibroids, the uterus may confer cardioprotective effects [9,10]. Whether removing the uterus (as opposed to the ovaries) is associated with long-term cardiometabolic outcomes remains unknown [11].

In approaching fibroid surgery, options include hysterectomy and myomectomy. Currently, long-term cardiometabolic outcomes of different fibroid surgeries are unknown, representing a critical knowledge gap.

This study had two objectives. First, we sought to quantify the incidence of adverse cardiometabolic outcomes among women with fibroids who underwent hysterectomy versus no surgery, myomectomy versus no surgery, and hysterectomy versus myomectomy. Second, to address the potential confounder of concomitant oophorectomy at the time of hysterectomy, we compared these same cardiometabolic outcomes between women who underwent hysterectomy with concomitant adnexal removal and age-matched, comorbidity-adjusted, adnexa-retaining controls who did not undergo surgery.

## 2. Materials and Methods

### 2.1. Study Design and Population

This retrospective cohort study utilized the Merative MarketScan^®^ Research Database (Ann Arbor, MI, USA) (formerly owned by IBM), a U.S. healthcare claims dataset, containing de-identified, individual-level data on healthcare utilization, expenditures, and outcomes [12].

**Figure 1:**
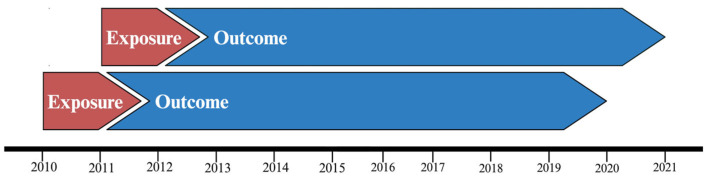
Timeline of study cohort.

We included women from MarketScan aged 18–55 in 2010 or 2011 with a diagnosis of fibroids (defined by ICD9 code 218.X in any inpatient or outpatient encounter) in 2010 or 2011, with continuous insurance enrollment for 10 years after their fibroid diagnosis. We identified whether patients in the cohort underwent uterine surgery and followed them for 10 years to determine their incidence of cardiometabolic disorders. This study followed the STROBE guidelines for observational studies [13]. Figure 1 illustrates the study timeline. This study was deemed non-human subject research by the Johns Hopkins University Institutional Review Board (IRB00229605). Informed consent was therefore not obtained.

### 2.2. Exposure

**Figure 2:**
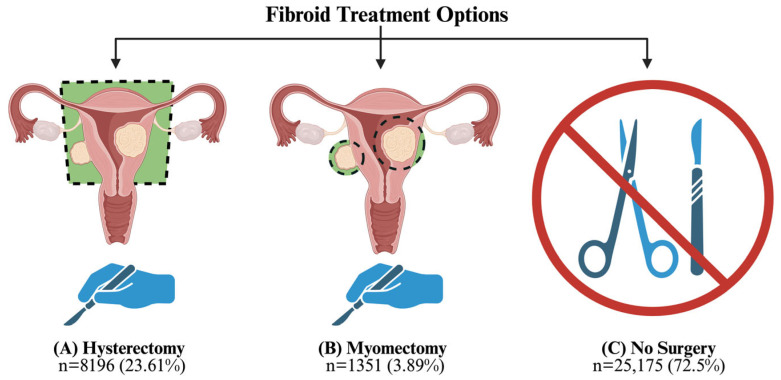
Cohort breakdown and size by intervention.

Patients were categorized into three comparison groups: hysterectomy, myomectomy, and no surgery (Figure 2). The “hysterectomy” group was defined by any inpatient or outpatient encounter within 365 days of their fibroid diagnosis, including selected procedures or diagnosis codes based on previously published literature [14,15]. The “myomectomy” group was defined as any inpatient or outpatient encounter within 365 days of their fibroid diagnosis, including selected procedures or diagnosis codes. The “no surgery” group was defined as having received neither surgery within 365 days of their fibroid diagnosis nor at any point in the 10-year follow-up. Any patient who received either surgery after the exposure period of 2010–2011 was excluded from analyses to ensure that all subjects were followed for 10 years after surgical exposure. We excluded patients who underwent multiple uterine surgeries outside the exposure period of 2010–2011. We treated patients who underwent myomectomy and hysterectomy during the exposure period (2010–2011) as having undergone hysterectomy, given that this treatment is functionally equivalent to those who solely underwent hysterectomy. We also excluded patients who underwent uterine artery embolization (UAE) (defined as any inpatient or outpatient encounter with the CPT code 37204) at any point and those who did not have continuous insurance enrollment for 10 years after their fibroid diagnosis.

### 2.3. Cardiometabolic Outcomes

Incident diagnoses of coronary artery disease (CAD), congestive heart failure (CHF), cardiac arrhythmia, stroke, hypertension, hyperlipidemia, type II diabetes (T2DM), and peripheral artery disease were defined using the ICD-9, ICD-10, and CPT codes specified in Appendix A based on previously published literature [16,17,18,19,20,21,22,23,24].

To evaluate the incidence of each of the eight cardiometabolic outcomes of interest, we removed all participants who already had a diagnosis of the outcomes of interest (i.e., prevalent cases) at the time of enrollment. In addition, as obesity is a modifier and risk factor for several cardiometabolic endpoints, we excluded all patients with a pre-existing obesity diagnosis. Subjects were characterized as incident cases if they had a diagnosis of the outcome of interest at any point in the 10 year follow up period. We defined incident diagnosis as the presence of any of the ICD-9, ICD-10, or CPT codes of interest—in both inpatient or outpatient settings—at any point after one year and up to and including 10 years from the exposure period (2010–2011).

### 2.4. Statistical Analysis

Baseline comparisons of geographic regions between groups were assessed using chi-square. For objective one, we created comorbidity-matched, age-adjusted multivariate logistic regression models for each cardiometabolic outcome of interest, comparing hysterectomy and myomectomy to the no surgery group. We then created comorbidity-matched, age-adjusted multivariate logistic regression models comparing hysterectomy to myomectomy. We report odds ratios and 95% confidence intervals for each cardiometabolic outcome. Crucially, we adjusted for age, as myomectomies are typically performed on younger patients compared to hysterectomies [25].

For objective two, we compared the risk of association of each cardiometabolic outcome for women who underwent adnexal removal at the time of hysterectomy to those who did not undergo surgery.

All analyses were performed using SAS v 9.4 (SAS Institute Inc., Cary, NC, USA). All our comparisons were sufficiently powered to detect differences at an alpha of 0.05.

## 3. Results

### 3.1. Cohort Selection and Demographics

**Figure 3:**
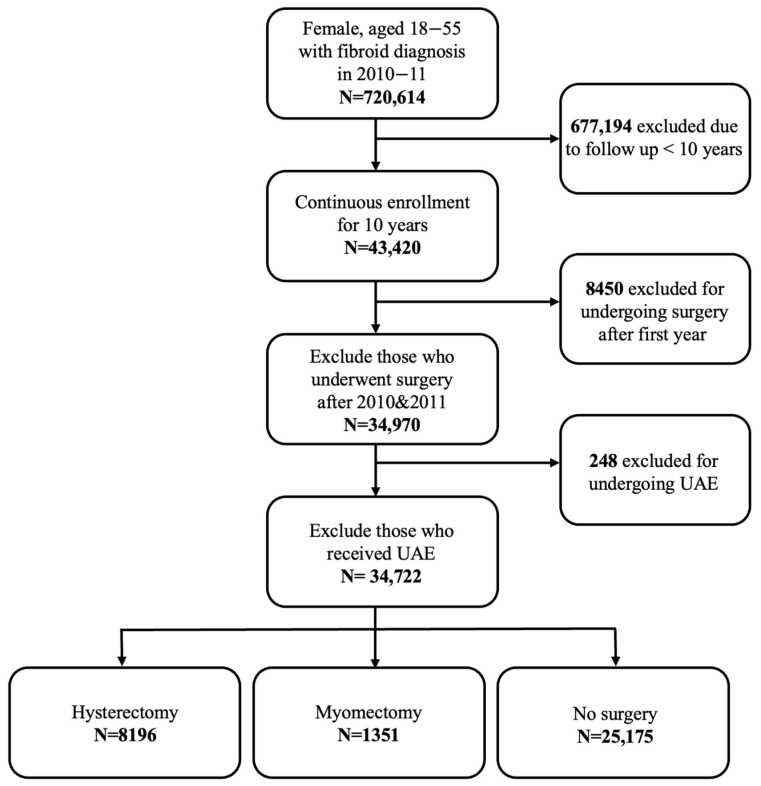
Study selection flowchart.

A total of 720,614 patients were identified with a fibroid diagnosis in 2010 or 2011. Of those, 43,420 patients had continuous enrollment for 10 years. A total of 8450 were excluded for undergoing surgery after the first year, and 248 were excluded for undergoing UAE, resulting in a total of 34,722 patients identified that met the study selection characteristics and were included in the analysis (Figure 3).

Our cohort included 8196 (23.61%) patients who underwent hysterectomy within 365 days of fibroid diagnosis (2010 or 2011), 1351 patients (3.89%) who underwent myomectomy within 365 days of fibroid diagnosis (2010 or 2011), and 25,175 (72.50%) patients who did not undergo either surgery. Most patients were from the Southern region of the United States. At baseline, patients who underwent myomectomy were significantly younger (median 39) than those who received no surgery (median 45) or hysterectomy (median 45) (Table 1).

### 3.2. Descriptive Statistics: Incident Cardiometabolic Case Numbers by Surgical Exposure

Of those who underwent hysterectomy, there were 644 incident cases of CAD, 187 incident cases of CHF, 919 incident cases of arrhythmia, 617 incident cases of stroke, 2624 incident cases of hypertension, 3803 incident cases of hyperlipidemia, 1417 incident cases of T2DM, and 362 incident cases of peripheral artery disease.

Of those who underwent myomectomy, there were 63 incident cases of CAD, 23 incident cases of CHF, 108 incident cases of cardiac arrhythmia, 56 incident cases of stroke, 326 incident cases of hypertension, 415 incident cases of hyperlipidemia, 158 incident cases of T2DM, and 36 incident cases of peripheral artery disease.

**Figure 4:**
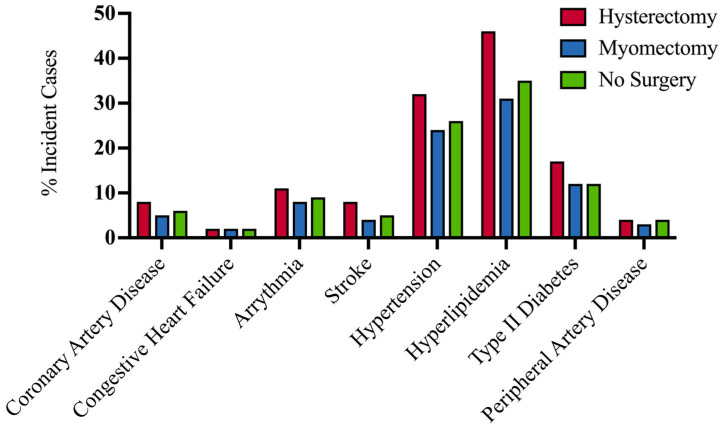
Percent of incident cases of cardiometabolic disorders by surgical exposure.

Of those who did not undergo surgery, there were 1479 incident cases of CAD, 454 incident cases of CHF, 2186 incident cases of cardiac arrhythmia, 1370 incident cases of stroke, 6587 incident cases of hypertension, 8876 incident cases of hyperlipidemia, 2981 incident cases of T2DM, and 892 incident cases of peripheral artery disease (Figure 4).

### 3.3. Hysterectomy Compared to No Surgery

After controlling for age, patients who underwent hysterectomy, compared to patients who did not undergo surgery, had higher odds of developing stroke (aOR = 1.11, *p* = 0.039), hypertension (aOR = 1.14, *p* < 0.0001), hyperlipidemia (aOR = 1.14, *p* < 0.0001), and T2DM (aOR = 1.20, *p* < 0.0001). There were no statistically significant differences between hysterectomy and no surgery in the odds of developing CAD, CHF, cardiac arrhythmia, and peripheral artery disease.

### 3.4. Myomectomy Compared to No Surgery

After the age adjustment, there were no statistically significant differences between myomectomy and no surgery for the eight outcomes.

### 3.5. Myomectomy Compared to Hysterectomy

After age adjustment, patients who underwent hysterectomy (vs. myomectomy) had increased odds of developing hyperlipidemia (OR = 1.26, *p* = 0.002). There were no statistically significant differences between hysterectomy and myomectomy regarding odds of developing CAD, CHF, arrhythmia, stroke, hypertension, T2DM, or peripheral artery disease.

### 3.6. Hysterectomy with Removal of Adnexa Compared to No Surgery

Women who underwent hysterectomy with concomitant adnexal removal had higher odds of developing CAD (OR = 1.21, *p* < 0.001), stroke (OR = 1.15, *p* = 0.024), hypertension (OR = 1.29, *p* < 0.001), hyperlipidemia (OR = 1.28, *p* < 0.001), and T2DM (OR = 1.37, *p* < 0.001) compared to those who did not undergo surgery (Table 2).

### 3.7. Hysterectomy with or Without Removal of Adnexal Structures

Among women who underwent hysterectomy, the odds of developing adverse cardiometabolic outcomes did not significantly differ between those with ovary-sparing procedures and those with oophorectomy (Table 3).

## 4. Discussion

We investigated the relationship between hysterectomy and subsequent development of adverse cardiometabolic outcomes among a large retrospective cohort of women with fibroids over a 10-year period. We further examined the impact of uterine-preserving surgery (myomectomy) on the development of cardiometabolic disorders when compared to both hysterectomy and women who underwent no surgery, as previous work suggested a potential link between fibroids and cardiometabolic risk [26,27,28]. To date, no study has examined long-term cardiometabolic outcomes in women undergoing hysterectomy for fibroids without concomitant oophorectomy to suggest whether the uterus itself may be associated with a cardioprotective benefit.

After adjusting for age matching for comorbidities, hysterectomy versus no surgery was associated with a significantly higher incidence of stroke, hypertension, hyperlipidemia, and T2DM. Compared to myomectomy and adjusting for age, hysterectomy was associated with a significantly increased incidence of hyperlipidemia. We replicated the finding that pre-menopausal removal of the adnexa at the time of hysterectomy is associated with worse long-term cardiometabolic outcomes compared to no surgery [29]. Importantly, however, we found that controlling for age, hysterectomy with removal of adnexa versus hysterectomy with retention of adnexa resulted in similar cardiometabolic outcomes, suggesting that hysterectomy may overshadow the effects of adnexa removal. Together, these results suggest that among women with fibroids, those who underwent hysterectomy—regardless of ovarian preservation status—had increased incidence of a multitude of adverse cardiometabolic disorders compared to both myomectomy and no surgery.

One could argue that the medical co-morbidities of women undergoing hysterectomy versus those choosing to forego surgery for fibroids may be different in ways that could potentially confound our results. To guard against this possibility, we chose to specifically focus on women with similar baseline medical history demographics. At time zero, all included women lacked the cardiometabolic outcomes of interest and had no diagnosis of obesity. The fact that those undergoing hysterectomy had worse long-term cardiometabolic outcomes suggests that the procedure itself, rather than patient factors, may have contributed to these outcomes.

The uterus is commonly thought of as a reservoir for reproduction that becomes vestigial after the reproductive years. However, evolving research suggests that the uterus may play a cardio-protective role independent of its reproductive function. The notion that the uterus is inert regarding cardiometabolic health and that its removal should bear no consequence on general health came under scrutiny in the HUNT2 study. Michelsen et al. examined a retrospective cohort of 47,000 Norwegian women who did not undergo surgery versus hysterectomy, versus bilateral oophorectomy without hysterectomy, for various indications. The investigators found that hysterectomy without bilateral oophorectomy was associated with increased all-cause and cardiovascular mortality, while oophorectomy was not associated with these outcomes, over an 18-year period [30]. Broni et al. studied 3367 female participants and found that hysterectomy alone was associated with a hazard ratio of 1.32 for the development of metabolic syndrome compared to no surgery, while hysterectomy with oophorectomy versus no surgery was associated with a comparable hazard ratio of 1.40 over a 10.5-year period [31]. Aside from the ovaries, the uterus itself may thus play a role in cardiometabolic health, through the so-called utero–cardiac axis [7].

We chose to focus on fibroids given that they represent the leading indication for hysterectomy. In the US, fibroids pose a public health burden, with substantial racial disparities in access to care and outcomes. A plethora of research has linked the presence of fibroids to cardiovascular disease risk factors, including hypertension and obesity [32,33]. While a causal link is still largely lacking, work from others and our laboratory has demonstrated a likely bidirectional relationship between uterine fibroids and cardiovascular disease [34,35,36].

### Strengths and Weaknesses of the Study

While the present study does not address causality, it does begin to clarify how either myomectomy or hysterectomy may be associated with variable cardiovascular risk profiles. Another advantage of narrowing our study’s focus to a single indication for surgery is to decrease heterogeneity of our sample in terms of patient factors and surgical factors. Strengths of our study also include the large sample size from geographically diverse regions of the US and long-term follow-up over a 10-year period, and the presence of both a surgical control with adnexal retention and a non-surgical control.

Our study also has limitations. The use of administrative databases allows for potential errors. Firstly, the accuracy of ICD and CPT codes is subject to clerical error and limited precision. Due to the de-identified nature of the dataset, there is a risk of unmeasured confounding as well as confounding by indication. We are limited by the data available in MarketScan. MarketScan data are collected for billing rather than research purposes and thus do not provide extensive demographic information. This database does not include race information, which would have helped elucidate whether factors such as systemic racism may moderate the relationship between hysterectomy/myomectomy and cardiometabolic outcomes. Next, MarketScan includes only those with private insurance; thus, findings may not be generalizable to populations on public insurance. Lastly, this was a retrospective study, so causal implications cannot be made.

## 5. Conclusions

Our study begins to address how uterine preservation may be associated with favorable cardiovascular outcomes over the lifespan. Potential mechanisms underlying this association remain to be determined using animal models and human physiological parameters (e.g., correlating hysterectomy with biomarkers of cardiovascular disease). Future studies may seek to control for the impact of post-oophorectomy use of hormone replacement therapy on cardiovascular outcomes.

## Figures and Tables

**Table 1:** Baseline demographics of included subjects.

	No Surgery	Myomectomy	Hysterectomy	*p*
	N = 25,175	N = 1351	N = 8196	
Age, median (IQR)	45 (40–49)	39 (35–45)	45 (41–48)	
N (%)				
Northeast	4369 (12.6)	200 (0.6)	691 (2.0)	<0.001
Northcentral	4781 (13.8)	199 (0.6)	1639 (4.7)	<0.001
South	13892 (40.0)	853 (2.4)	5218 (15.0)	<0.001
West	2065 (6.0)	93 (0.2)	630 (1.8)	0.03
Unknown	68 (0.2)	6 (0.0)	18 (0.1)	0.15

IQR interquartile range.

**Table 2:** Cardiometabolic disease incidence in patients with fibroids who underwent hysterectomy with adnexa removal compared to patients who did not undergo surgery.

	OR (95% CI)	*p*
Cardiac Arrhythmia	1.05 (0.95–1.17)	*p* = 0.32
Peripheral Artery Disease	1.03 (0.88–1.20)	*p* = 0.72
Coronary Artery Disease	1.21 (1.08–1.36)	*p* < 0.001
Congestive Heart Failure	1.03 (0.83–1.23)	*p* = 0.77
Stroke	1.15 (1.02–1.30)	*p* = 0.024
Type II Diabetes	1.37 (1.25–1.50)	*p* < 0.001
Hyperlipidemia	1.28 (1.18–1.38)	*p* < 0.001
Hypertension	1.29 (1.19–1.40)	*p* < 0.001

**Table 3:** Cardiometabolic disease incidence in patients with fibroids who underwent hysterectomy with adnexa retention compared to patients with removal of adnexa at time of hysterectomy.

	OR (95% CI)	*p*
Cardiac Arrhythmia	0.69 (0.47–1.00)	*p* = 0.06
Peripheral Artery Disease	1.78 (0.94–3.36)	*p* = 0.08
Coronary Artery Disease	0.99 (0.61–1.60)	*p* = 0.96
Congestive Heart Failure	1.14 (0.53–2.44)	*p* = 0.74
Stroke	1.35 (0.85–2.16)	*p* = 0.20
Type II Diabetes	1.38 (0.98–1.93)	*p* = 0.06
Hyperlipidemia	1.24 (0.94–1.63)	*p* = 0.12
Hypertension	1.06 (0.79–1.4)	*p* = 0.72

## Data Availability

Data is available upon request to the corresponding author.

## Abbreviations

The following abbreviations are used in this manuscript:

ICD	International Classification of Diseases
CPT	Current Procedural Terminology
UAE	Uterine artery embolization
T2DM	Type II diabetes
CHF	Congestive heart failure
CAD	Coronary artery disease
U.S.	United States
aOR	Age-adjusted odds ratio
OR	Odds ratio
IQR	Interquartile Range
SAS	Statistical Analysis System
STROBE	Strengthening the Reporting of Observational Studies in Epidemiology

## Appendix A

**Table A1:** ICD9, ICD10, and CPT codes used in analysis.

Variable	CPT	ICD9	ICD10
Uterine fibroids		218	D25
Hysterectomy	58294, 58293, 58541, 58275, 58280, 58290, 58267, 58270, 58260, 58570, 58543, 58550, 58572, 58553, 58150, 58292, 58152, 58180, 58542, 58544, 58552, 58554, 58571, 58573, 58262, 58263, 58291	68.39, 68.49, 68.31, 68.41, 68.51, 68.4, 68.59	
Hysterectomy—presumed ovarian preserving	58294, 58293, 58541, 58275, 58280, 58290, 58267, 58270, 58260, 58570, 58543, 58550, 58572, 58553		
Hysterectomy—with presumed oophorectomy	58150, 58292, 58152, 58180, 58542, 58544, 58552, 58554, 58571, 58573, 58262, 58263, 58291		
Myomectomy	58140–58146 or 58545–58546	68.19, 68.29	
Uterine artery embolization	37204		
Coronary artery disease	92920, 92921, 92924, 92925, 92928, 92929, 92933, 92920, 92921, 92924, 92925, 92928, 92929, 92933, 92934, 92937, 92938, 92941, 92973, 92975, 92977, 93454, 93455, 93456, 93457, 93458, 93459, 93460, 93461	410, 411, 412, 413, 414	I20, I21, I22, I23, I24, I25
Congestive heart failure		428	I50
Cardiac arrhythmias		427	I46, I47, I48, I49
Stroke	37195, 37201, 37202	430, 431, 432, 433, 434, 435, 436, 437, 438	I60, I61, I62, I63, I64, I65, I66, I67, I68, I69
Hypertension		401, 402, 403, 404, 405	I10, I11, I12, I13, I15
Hyperlipidemia		272	E780, E781, E782, E783, E784, E785
Diabetes		250	E10, E11, E12, E13, E14
Obesity		278	E66–E68
Peripheral artery disease		440, 441, 442, 443, 444, 445	I70
